# IDH Mutations in Chondrosarcoma Correlate with Patient Survival in De-Differentiated but Not Conventional Subtypes

**DOI:** 10.3390/jcm14093058

**Published:** 2025-04-29

**Authors:** Jay Swayambunathan, Paula Viza Gomes, Robert Valente Childers-Quiñones, Nicole Levine, Julia Visgauss

**Affiliations:** 1 Department of Orthopaedic Surgery, Duke University, Durham, NC 27710, USA; 2 University of Arizona College of Medicine, Tucson, AZ 85724, USA

**Keywords:** chondrosarcoma, isocitrate dehydrogenase, IDH, IDH1, IDH2

## Abstract

**Background:** Chondrosarcoma is the second most common bone tumor in adults with an average incidence of 0.1–0.3 individuals per 100,000 per year. These tumors are often resistant to chemotherapy and radiation, and surgical excision is a mainstay of current treatment. However, survival in the setting of metastatic disease is still poor, and research is needed to identify prognostic biomarkers and potential therapeutic targets. Several studies have examined the role of IDH mutations in chondrosarcoma, but the results vary widely. The goal of this analysis was to aggregate individual patient data from these studies and conduct a high-powered analysis of the impact of IDH mutations on survival outcomes in chondrosarcoma. **Methods:** Chondrosarcoma studies that included data on the IDH mutation status of tumors were queried, and the individual datasets reporting patient and tumor variables were extracted. The data from these studies were added to the internal dataset from the authors’ home institution. Two-sample tests for equality of proportions were used to assess the distribution of sample characteristics between groups. Univariate Kaplan–Meier (KM) curves and multivariate Cox Proportional Hazards (CPH) models were used to assess the relationship between tumor IDH mutations and five and ten-year patient overall survival (OS). **Results:** The final cohort included 1152 patients sourced from 21 studies and the authors’ internal dataset. IDH mutations were more common in higher grade tumors and were more likely to be found in individuals over 60 years old. Patients with IDH mutant tumors had shorter five-year OS in univariate KM analysis when analyzing all chondrosarcomas combined. However, multivariate CPH models accounting for age and tumor grade, found that the effect of IDH mutation was isolated to patients with dedifferentiated tumors only. Patients with IDH mutant dedifferentiated tumors displayed significantly shorter five-year OS (HR: 1.99, *p* = 0.02) relative to patients with IDH wild-type (WT) dedifferentiated tumors. The primary predictor of five-year OS in the conventional chondrosarcoma cohort was tumor grade, regardless of IDH mutation status (HR: 2.72, *p* < 0.005). **Discussion:** IDH mutations are relatively common in cartilaginous neoplasms (including benign tumors), with the literature reporting rates as high as 50% in chondrosarcomas. Prior studies have investigated the link between IDH1/2 mutation status, tumor grade and overall survival, with mixed results on the effect of IDH mutation on survival. Vuong et al. performed a meta-analysis in 2021 and found that IDH mutation was associated with older patient age, larger tumor size, higher tumor grade, and increased risk of death compared to WT tumors. Our analysis, which builds on the Vuong et al. study, indicates that IDH status itself is not independently predictive of overall survival in conventional chondrosarcoma, however, it does correlate with survival in dedifferentiated tumors. Further analysis is needed to investigate the potential correlation of IDH mutations in higher grade tumors and patients of older age.

## 1. Introduction

Chondrosarcomas are the second most common bone tumors in adults worldwide, exhibiting an average annual incidence of 0.1–0.3 cases per 100,000 individuals [1]. Typically diagnosed between the third and sixth decades of life, these tumors are grouped into several subtypes based on the 2020 World Health Organization (WHO) guidelines [2]. These subtypes include, but are not limited to, atypical cartilaginous tumors, conventional chondrosarcomas, and dedifferentiated chondrosarcomas [2].

Patients diagnosed with low-grade (grade 1) chondrosarcomas exhibit a 10-year survival rate ranging from 88% to 95% and rarely develop metastatic disease [3]. However, intermediate and high-grade tumors are associated with worse prognosis and have 10-year survival rates of 58–86% and 26–55%, respectively [3]. These cases warrant more aggressive treatment involving wide local resection and carry a metastatic risk reported between 32–70% depending on the grade [4].

Despite the WHO guidelines in describing tumors and predicting their behavior, the differences between tumor grades in chondrosarcoma can be subtle, leading to high interobserver variability in grading among pathologists [5]. As such, prognostication based on grading alone is limited and the integration of additional factors (such as tumor size, extra-osseus extension, and location, etc.) have become an important part of prognostic evaluation. However, prognostication based on genetic and molecular characterization of these tumors remains limited without current formal clinical recommendations. Moreover, the general resistance of chondrosarcomas to conventional therapies like radiation and chemotherapy has further highlighted the need to identify subclasses of tumors that may respond to novel targeted therapeutics.

The isocitrate dehydrogenase (IDH) gene is one such target, and research suggests that IDH mutations may be present in up to 50% of chondrosarcomas and are involved in the pathogenesis of several other malignancies (e.g., gliomas/glioblastomas, cholangiocarcinomas, and acute myeloid leukemia) [6,7]. Mutations in IDH1/2 increase levels of 2-hydroxyglutarate (2HG), a metabolite that has been shown to impact epigenetic regulation as well as cellular differentiation and metabolism [6]. There is also evidence to suggest that 2HG impairs immune surveillance by altering T cell metabolism [8]. However, the effects of IDH mutations are likely malignancy-specific, and highly dependent on context and timing [6]. As such, refining our understanding of the impact of IDH mutations in different malignancies is crucial for understanding disease biology and designing safe and effective therapeutics.

While the presence of IDH mutations in chondrosarcoma is well documented, the correlation between IDH mutation status and overall survival, metastatic disease, and local recurrence is not well understood. Some studies suggest that IDH mutations have a clear negative effect on overall survival and portend poor patient prognosis [7,9,10,11,12]. Other studies suggest that IDH mutations have little to no impact in chondrosarcoma prognosis [13,14,15]. In 2021, Vuong et al. published a meta-analysis incorporating data from 14 studies (488 patients) and found that IDH mutations had a negative correlation with overall patient survival [11]. Additionally, they found that IDH mutations correlated with advanced patient age, higher tumor grade, larger tumor diameter, and specific tumor sites, each of which are variables that have also independently been shown to correlate with patient survival [11]. In recent years, IDH mutation testing has become more routine in chondrosarcoma, and a growing number of new publications with this data are now accessible. The amount of available patient data has more than doubled and, given the conflicting literature evaluating the correlation between IDH mutation status and patient outcomes in chondrosarcoma, we elected to investigate this rigorously in a large cohort. This analysis seeks to aggregate data from all studies published to date with known IDH mutation status and oncologic outcomes, and isolate confounding variables to conduct a high-powered analysis of IDH status and its correlation to disease progression and survival in patients with chondrosarcoma.

## 2. Methods

### 2.1. Institutional Review Board (IRB) Approval

IRB approval was obtained for the use of internal patient data from the authors’ home institution. IRB approval was not necessary for individual patient data sourced from published works.

### 2.2. Internal Patient Data

The records of patients treated for chondrosarcoma at Duke University Medical Center in Durham, North Carolina, between 2005 and 2023 were retrieved. Patient data were incorporated into the analysis if survival data and IDH mutation status were available. Twenty-one patient records met these criteria, and the data from these individuals were included in the analysis

### 2.3. Literature Review

The initial dataset consisted of individual patient data extracted from articles utilized by Vuong et al. in their original meta-analysis [11]. PubMed and ScienceDirect databases were then queried to search for articles published between November 2020 and March 2023, with the aim of including articles not represented in their original dataset. The following query term was used to identify relevant articles:

chondrosarcoma AND (IDH1 OR IDH2 OR IDH OR isocitrate dehydrogenase OR IDH1/2)

**Figure 1:**
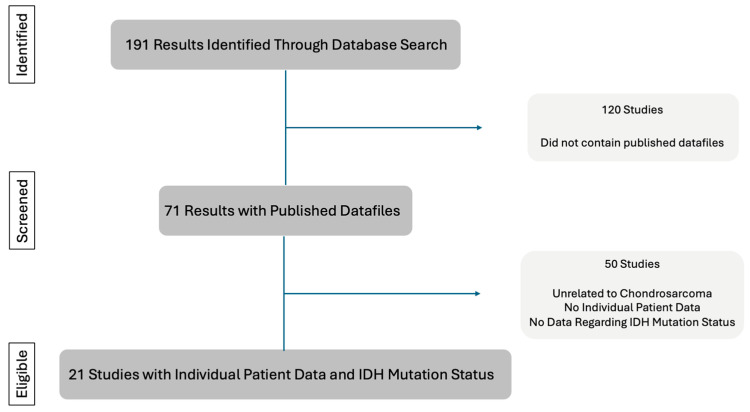
Analysis Flowchart. The process of collecting, screening, and identifying eligible studies.

Studies were included if they contained individual patient data and IDH mutation data for chondrosarcoma patients. The patient data extracted from each study included: authors, year of publication, patient demographics, tumor grade, tumor site, overall survival [OS] status, OS time, presence of any IDH mutation, IDH1 mutation, IDH2 mutation, IDH mutation type, metastasis, and local recurrence. Figure 1 outlines the process used to identify and filter eligible studies. Table 1 provides a breakdown of the studies used and the data types sourced from each study. None of the included studies were designed to compare treatment effects.

### 2.4. Statistical Analysis

All statistical analysis for this study was done in Python 3.10.13 (Python Software Foundation, Wilmington, DL, USA). Packages used include Pandas 2.2.1, Numpy 1.26.4, SciKit Learn 1.4.1, matplotlib 3.8, lifelines 0.28.0, and Kaplan-–Meier (© Erdogan Taskesen, 2020). A two-sided *p* value < 0.05 was considered significant. Categorical variables were compared between groups using a two-sample Z-test for proportions. Continuous variables were compared using a standard two-sample Z test. The distribution of variables was assessed through visual inspection of histograms prior to their use in modeling. If studies did not report the variable of interest, patients from these studies were omitted from subset analysis.

Chondrosarcoma patients were grouped into conventional (grades 1, 2, 3) or dedifferentiated (designated as dedifferentiated and/or grade 4) based on tumor grade recorded in the original study. Some studies reported both grade and dedifferentiation status, while others reported only dedifferentiation status. As such, tumors displaying any dedifferentiation were grouped into a “dedifferentiated” category regardless of associated lower-grade component. Univariate Kaplan–Meier (KM) curves and multivariate Cox Proportional Hazards (CPH) models were constructed to visualize the association between categorical variables and five-year overall survival (OS). Tumor size and tumor location were not available for all patients, and as such, only patient age, sex, tumor grade, and IDH status were included in multivariate modeling. Studies reporting tumor characteristics, but lacking follow-up status or time were excluded from the survival analysis.

## 3. Results

The final sample contained raw data from 1152 patients sourced from 21 studies, as well as the authors’ internal cohort of 21 patients. Most patients fell into the 41–60 and 61–80-year-old age groups. Grade 1 (300, 26.7%), grade 2 (503, 44.8%), grade 3 (101, 9.0%), and dedifferentiated tumors (220, 19.6%) were well represented in the set. The dataset was roughly balanced between IDH wild-type (WT) (545, 47.19%) and IDH mutant (610, 52.81%) tumors. IDH WT status was only assigned to patients with successful genetic testing that detected no IDH 1 or IDH 2 mutations. No information was available regarding the percentage of patient samples that could not be analyzed due to technical issues or inadequate sample quality.

Patients with metastatic disease (245, 34.22%) and locally recurrent disease (98, 35.51%) were well represented. Appendicular tumors (556, 54.09%) were slightly more common than axial tumors (472, 45.91%). The femur was the most common tumor site (254, 24.71%), and just over half of tumors were less than 10 cm in diameter (431, 58.9%). In terms of final follow up status, patients were categorized as no evidence of disease (NED) (447, 60.41%), alive with disease (AWD) (47, 6.35%), dead of disease (DOD) (193, 26.08%), and dead of other cause/unknown (DO) (53, 7.16%). It is important to note that only a few studies reported patients who died from other causes, and most of these patients were represented by a single study [9]. A detailed breakdown of overall patient and tumor characteristics are provided in Table 2 and Table 3, respectively.

**Figure 2:**
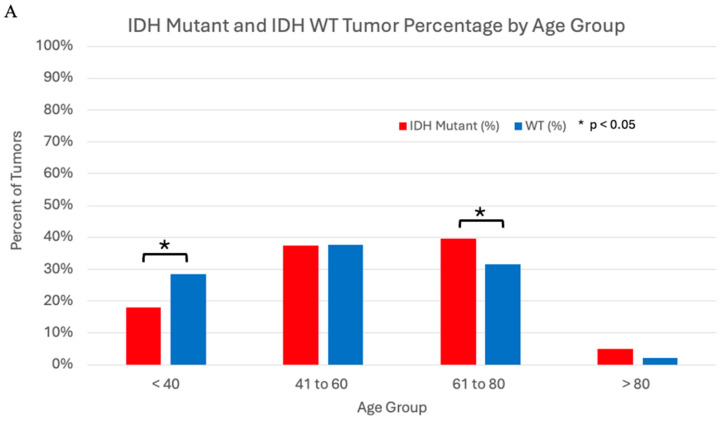

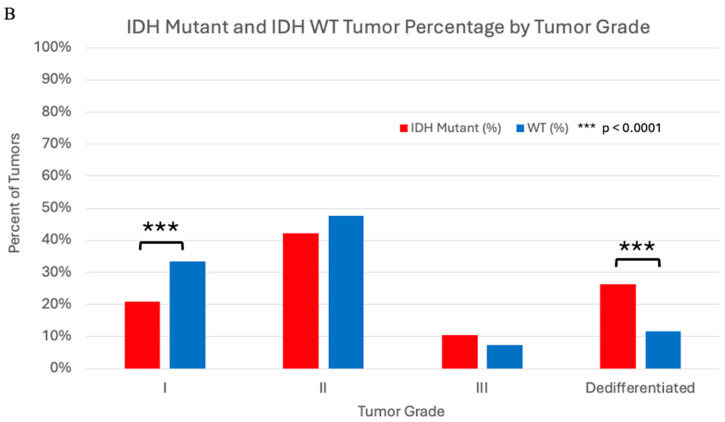
IDH Mutation Percentage by Age Group (**A**) and Tumor Grade (**B**). Correlation between patient age and IDH mutation status as well as tumor grade and IDH mutation status. Patients with IDH mutant tumors were more likely to be in the 61- to 80-year-old age range (39.53% vs. 31.43%, *p* = 0.0354) and less likely to fall in the less-than-40 age range (17.94% vs. 28.57%, *p* = 0.0018) than patients with WT tumors. IDH mutant tumors were more likely to display dedifferentiation than WT tumors (26.42% vs. 11.66%, *p* < 0.0001) and less likely to fall into the grade 1 category (20.90% vs. 33.46%, *p* < 0.0001).

Table 4 offers a breakdown of the sample based on IDH WT status and the presence of any IDH mutation (IDH1 and/or IDH2). IDH mutant tumors were more likely to occur in patients over 60 years of age, and less likely to occur in patients under 40 years of age. Additionally, IDH mutations were more commonly seen in dedifferentiated than conventional tumors (26.42% vs. 11.66%, *p* < 0.0001). The trending correlation between tumor grade and patient age with IDH mutation status can be better visualized in Figure 2. Patients with IDH mutant tumors were slightly more likely to have died of disease at final follow up compared to their WT counterparts (28.72% vs. 21.09%, *p* = 0.0246). IDH mutant tumors were less likely to be associated with local recurrences than WT tumors (28.97% vs. 42.75%, *p* = 0.0169), and IDH mutant tumors were more likely to be found at appendicular sites than WT tumors (68.29% vs. 37.42%, *p*= 0.0312).

With regard to time to metastasis and time to local recurrence, these data were only available for a minority of patients (41 and 63 patients, respectively). We completed an analysis using only data from these patients and found no significant difference between IDH mutant and IDH WT tumors in average time to metastasis (26.88 months vs. 33.22 months, respectively, *p* = 0.6275) or in average time to local recurrence (37.7410 months vs. 32.2833 months, respectively, *p* = 0.5777).

Table 5 provides a breakdown of the IDH mutant sample between IDH 1 and IDH 2 mutant tumors. These data were not available in all studies, which limited our ability to include this variable in subsequent analyses. Patients with lower grade tumors (grade 1 and grade 2 chondrosarcoma) were more likely to have IDH 1 mutant tumors (23.97% vs. 11.38% in grade 1 (*p* = 0.0024), 44.06% vs. 32.53% in grade 2 (*p* = 0.021), respectively), while patients with dedifferentiated tumors were more likely to have IDH 2 mutant tumors (22.89% vs. 43.09%, *p* < 0.0001). Patients with appendicular disease were more likely to have IDH 2 mutant tumors (62.79% vs. 89.52%, *p* < 0.0001) and patients with axial disease were more likely to have IDH 1 mutant tumors (37.21% vs. 10.48%, *p* < 0.0001).

**Figure 3:**
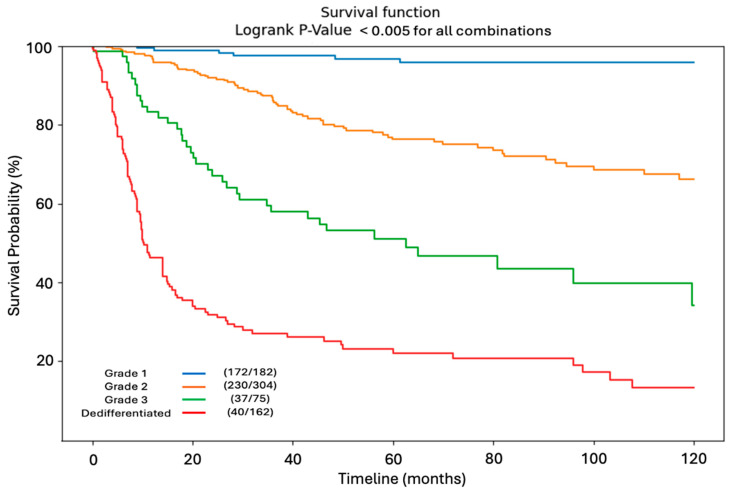
Kaplan–Meier Curve. Ten year overall survival by tumor grade. The ten-year univariate KM survival curve stratified by tumor grade. Survival decreases significantly as tumor grade increases, with the poorest survival dynamics seen in patients with dedifferentiated tumors. The number of surviving patients at final endpoint relative to total number of starting patients is provided in the legend (e.g., Grade 1—172/182 = 172 surviving patients out of 182 total initial patients in the group).

Figure 3 depicts the ten-year univariate KM survival curve stratified by tumor grade. Survival decreases significantly as tumor grade increases, with the poorest survival dynamics seen in patients with dedifferentiated tumors. The number of surviving patients at final endpoint relative to total number of starting patients is provided in the legend (e.g., Grade 1—172/182 = 172 surviving patients out of 182 total initial patients in the group).

**Figure 4:**
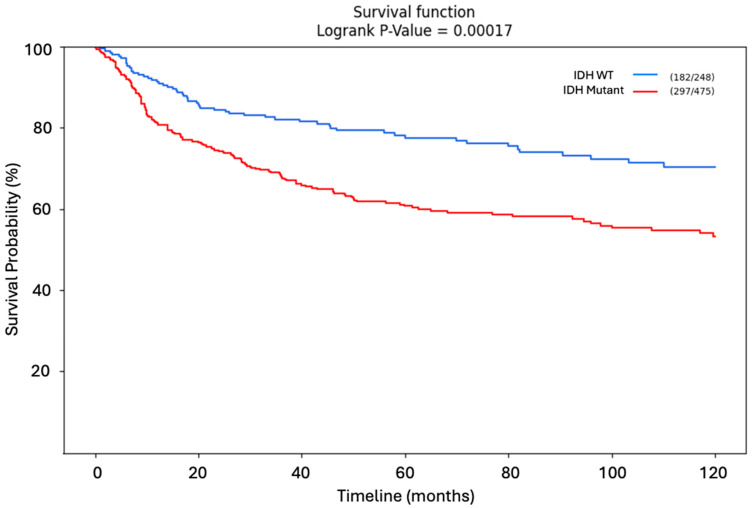
Kaplan–Meier Curve. Ten-year overall survival in chondrosarcoma by IDH status. Univariate KM analysis of ten-year OS in the total chondrosarcoma sample (both dedifferentiated and conventional) revealed decreased survival in patients with IDH mutant tumors relative to IDH WT tumors. This separation was statistically significant (*p* = 0.0002).

Univariate KM analysis of ten-year OS in the full chondrosarcoma sample (both dedifferentiated and conventional) revealed decreased survival in patients with IDH mutant tumors relative to IDH WT tumors. This separation was statistically significant (*p* = 0.0002). These results are provided in Figure 4.

**Figure 5:**
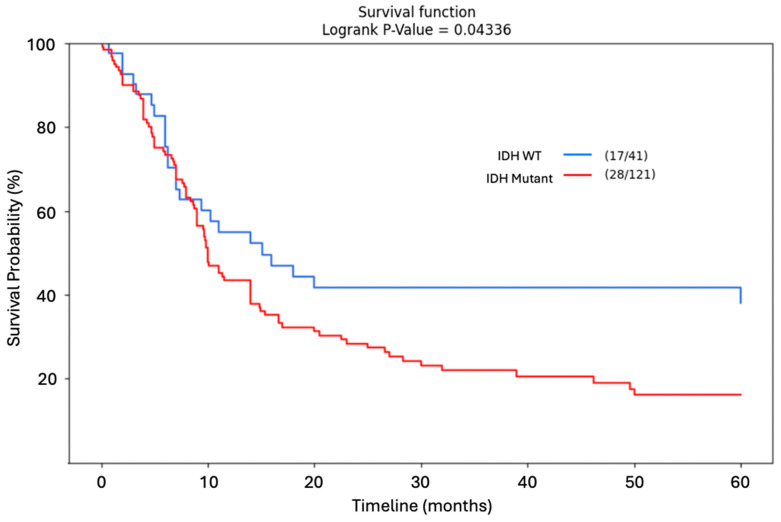
Kaplan–Meier Curve. Five-year overall survival in dedifferentiated chondrosarcoma by IDH status. A KM analysis confined to dedifferentiated chondrosarcomas unveiled a significant difference in five-year OS between IDH mutant and IDH WT patients with IDH WT patients displaying a higher survival proportion at the final endpoint (*p* = 0.0434). The low proportion of surviving patients after five years precluded a ten-year analysis in this case.

A similar analysis confined to dedifferentiated chondrosarcomas unveiled a significant difference in five-year OS between IDH mutant and IDH WT patients with IDH WT patients displaying a higher survival proportion at the final endpoint (*p* = 0.0434). The low proportion of surviving patients after five years precluded a ten-year analysis in this case. This result is presented in Figure 5.

Next, a multivariate analysis was performed with all variables available from the compiled studies (including patient age, sex, tumor grade, and IDH mutation status). Table 6 contains the results of multivariate CPH models relating to five-year OS. Patient sex was not a significant predictor of survival and is omitted from the results presented here. IDH mutation status correlated with OS in the full cohort (HR: 1.6, *p* = 0.04), however, this was driven by the dedifferentiated cohort. When analyzed separately, there was no correlation of IDH mutation status in the conventional chondrosarcoma group, but rather only in the dedifferentiated tumors, which was statistically significant (HR: 2.00, *p* = 0.02). Patient age (HR: 1.04, *p* < 0.005) and tumor grade (HR: 2.72, *p* < 0.005) were the only significant predictors of survival in patients with conventional chondrosarcoma.

When evaluating the demographic data of these tumors, it is clear that there is a very small percentage of tumors classified as grade 3 without a dedifferentiated component. While it is possible that this category of tumors is truly small, it may also highlight the histological limitations of using conventional numerical grading schemes for these tumors. For this reason, we conducted additional analysis with the designation of “low-grade” (grade 1) vs. “high-grade” (grade 2 or 3) for the conventional chondrosarcomas. Table 7 contains the results of multivariate CPH models relating patient age, patient sex, tumor grade (high vs. low) and IDH status with five-year OS. Again, patient sex was not a significant predictor of survival in this analysis and is omitted from the results presented here. Interestingly, the effect of IDH mutation in this analysis became even less apparent in the overall cohort and remained non-contributory in the conventional cohort. The presence of IDH mutation was only a significant predictor of OS in the dedifferentiated chondrosarcoma population (HR: 1.99, *p* = 0.02). As one might imagine, grading became a much more powerful predictor of survival in conventional chondrosarcoma when high-grade vs. low-grade designation was used (HR: 11.2, *p* = 0.02).

## 4. Discussion

Several studies have identified factors associated with chondrosarcoma prognosis. The development of metastatic disease is the most significant predictor of survival in chondrosarcoma patients, correlating with higher tumor grade, larger tumor size, and axial location [30]. Other studies have found that patient age, histological type, tumor grade, and AJCC stage have significant effects on post-surgical survival [31]. Despite this knowledge, prognostication remains a challenge, prompting researchers to explore additional factors that can enhance our understanding of chondrosarcoma behavior. Furthermore, given the general resistance of chondrosarcoma to chemotherapy and radiation, there is a concerted effort to identify novel targeted therapies. IDH is one such target and clearly defining the effect that IDH mutations play in chondrosarcoma is a major step towards producing effective, evidence-based treatments for patients with this condition. Currently, clinical trials assessing the safety and efficacy of mutant IDH 1/2 inhibitors are underway but include heterogenous tumor populations and variable efficacy [32]. Continuing to develop our understanding of the importance of IDH in chondrosarcoma will allow us to optimize the benefits of such treatments in the future.

IDH mutations are relatively common in cartilage forming tumors, with the literature reporting rates as high as 50% in chondrosarcomas [7]. Xenograft studies on 10T cells found that IDH2 mutant cells generated palpable tumors 20 days post-injection, whereas WT cells did not, indicating a potential role of IDH mutation in disease susceptibility and tumorigenesis [33]. In the same study, IDH2 mutant tumor histology and immunohistochemistry resembled that of poorly differentiated sarcomas, suggesting that IDH mutation may influence disease progression and dedifferentiation [33]. These findings can potentially be explained by the fact that IDH mutation alters the production of 2-hydroxyglutarate (2HG), a pro-oncogenic metabolite. 2HG affects histone methylation and induces the formation of abnormal DNA [7,16]. It may also impair immune surveillance by altering T cell metabolism and the tumor microenvironment [8]. Such work provides important insight into biochemical mechanisms that can be altered because of IDH mutation, but the correlation in behavior between naturally occurring IDH mutations in human chondrosarcomas versus artificially induced IDH mutations in murine models is unclear. Research also suggests that the role of IDH mutations varies between malignancies, so chondrosarcoma-specific research is required to uncover the precise role of IDH mutation in pathogenesis [6].

The existing literature has investigated the link between IDH1/2 mutation status, tumor grade, and overall survival, but findings across the literature have been varied. Some studies report a significant negative impact of IDH mutations on patient survival [7,9,10,11,12]. Other studies found no such effect independently, suggesting that IDH status may be correlated with dedifferentiation/higher tumor grade which are the real drivers of patient outcomes [13,14,17]. The basis of our study stemmed from a meta-analysis published in 2021, which synthesized data from 14 studies encompassing 488 patients. It revealed that IDH mutations had a negative impact on OS, and correlated with advanced patient age, higher tumor grade, larger tumor size, and specific tumor sites [11]. Our study aimed to build on these results, incorporating data published in the interim and using multivariate techniques to account for these potential confounding variables, in order to make a more definitive statement about the effect of IDH mutations independently on patient outcomes in chondrosarcoma.

Univariate survival analysis showed a significant relationship between IDH mutation status and five-year OS in all chondrosarcomas, however, multivariate analysis standardized for patient age and tumor grade indicated that IDH mutations independently negatively impact survival among patients with dedifferentiated tumors only. Conventional tumors did not show an association between IDH mutation and OS, and the most significant predictor of survival in this population remains tumor grade (either numerical (1, 2, 3) or low vs. high designation). Given that IDH mutations were more prevalent in higher grade tumors, the effect of IDH mutation status in univariate analysis is likely driven by the effect of grade in conventional tumors.

These data suggest that, while IDH mutation status may be a marker for worse prognosis in the already highly aggressive dedifferentiated subsets, it does not support the predictive nature of IDH mutation status alone in conventional chondrosarcoma. However, like the Vuong, et al. study, our cohort did demonstrate a correlation between the presence of an IDH mutation with tumor grade and patient age; with increasing patient age and tumor grade correlating with an increased likelihood of having an IDH mutation [11]. It is unclear whether this is the result of differences in the underlying biology of these tumors or the result of age-related derangements of cellular metabolism in older patients with chondrosarcoma. It certainly provides evidence for the need for further research in this area.

When examining rates of local recurrence, IDH mutant tumors appear to be associated with lower risk of local recurrence. While the true effect of this is unclear, we suspect two potential reasons for this. First, IDH WT tumors were more likely to fall into the Grade 1 category, which are often treated with local curettage rather wide excision. This may lead to an iatrogenic elevation in local recurrence rate in this population. Additionally, patients with IDH mutant tumors were also more likely to have high-grade/dedifferentiated tumors. These patients display shorter overall survival which may impact the probability of developing a local recurrence during the life span of these individuals. Our analysis did not detect a difference between IDH mutant and IDH WT tumors with regards to metastatic disease, however, it is important to note that most studies reported overall survival but lacked data on time to metastasis or time to disease recurrence, which limited this evaluation.

Overall, our analysis suggests that IDH mutations have an independent impact on survival in patients with dedifferentiated chondrosarcoma. When accounting for patient age and tumor grade, we found that tumor grade was a more significant predictor of patient survival in conventional chondrosarcoma than IDH status. Additional time-to-event analyses regarding metastasis and local recurrence are needed to conclusively demonstrate an association between IDH mutation and these outcomes.

The efficacy of IDH1 inhibitors in chondrosarcoma are currently being assessed in clinical trials, though populations are heterogeneous or limited to only patients with conventional chondrosarcoma [34]. The exclusion of dedifferentiated chondrosarcoma from these studies may be due to established chemotherapy guidelines in dedifferentiated subtypes [35], and/or the absence of a standard treatment for conventional chondrosarcoma. However, our data from this study would suggest that patients with dedifferentiated chondrosarcoma may serve to benefit the most, and including patients with only conventional chondrosarcoma may limit the ability to see an effect. Further investigation and consideration of chondrosarcoma subtype should be made when analyzing efficacy and designing future clinical trials with IDH inhibitors.

Our study has several limitations. Data were sourced from multiple studies conducted between 2011 and 2024 each reporting different metrics on different patient populations. Several studies did not report patient age, patient sex, tumor size, or tumor location so data from these studies were excluded from multivariate modeling. Some studies did not report IDH genotype (only IDH mutation or WT) which limited the power of the IDH1 vs. IDH2 mutant analyses. Our ability to analyze the effects of IDH1 vs. IDH2 mutation status and patient survival was also limited by class imbalance within the dataset. IDH1 mutations were seen with approximately four-times greater frequency than IDH2 mutations in our sample (472 patients vs. 125 patients, respectively). Most of the studies used to compile the dataset for this work did not contain information regarding the treatment protocols that patients received, and, as such, we were not able to standardize for treatment effects. However, trials of IDH inhibitors are recent and include very small subsets of chondrosarcoma patients, so we believe that the inability to account for this is not a significant limitation given the overall size of our dataset. Finally, most studies did not report time to metastasis or time to local recurrence which limited the temporal resolution and power of this analysis.

## 5. Conclusions

Our analysis indicates that IDH mutation status correlates with overall survival in patients with dedifferentiated chondrosarcoma. Tumor grade is the most significant predictor of overall survival in patients with conventional chondrosarcoma, and while IDH mutation did correlate with survival on univariate analysis, this was driven by the effect of grade and was not significant on multivariate analysis. Further time-to-event analysis is required to assess the association between IDH status and rates of metastatic and locally recurrent disease.

## Figures and Tables

**Table 1:** Data Sourced from Included Studies. A list of studies from which raw patient data were obtained, including a breakdown of variables sourced from each. Variables which are present are highlighted in light gray while absent variables are highlighted in dark gray. The final sample contained 1152 patients sourced from 21 studies as well as the authors’ internal cohort [6,7,9,10,11,12,13,14,15,16,17,18,19,20,21,22,23,24,25,26,27,28,29].

Study	Year	Patient Age	Patient Sex	Tumor Grade	Tumor Site	Tumor Size	Overall Survival Time	Overall Survival Status	IDH Mutation Status	Metastasis	Local Recurrence
Trovarelli	2024	Y	Y	Y	Y	N	Y	Y	Y	Y	Y
Nakagawa	2022	Y	Y	Y	Y	N	Y	Y	Y	Y	N
Abdulfatah	2023	Y	Y	Y	Y	Y	Y	Y	Y	Y	Y
Lyskjaer	2021	Y	Y	Y	Y	Y	Y	Y	Y	Y	Y
You	2023	Y	Y	Y	Y	N	N	N	Y	N	Y
Cross	2022	N	Y	Y	Y	Y	Y	Y	Y	Y	N
Pacheco	2022	Y	Y	Y	Y	Y	Y	Y	Y	Y	Y
Asioli	2020	Y	Y	Y	Y	N	N	N	Y	N	N
Amary	2011	Y	Y	Y	Y	Y	Y	Y	Y	Y	Y
Arai	2012	Y	Y	N	Y	N	N	N	Y	N	N
Chen	2017	Y	Y	Y	Y	Y	N	N	Y	N	N
Zhu	2020	Y	Y	Y	Y	Y	N	N	Y	Y	N
Gambarotti	2020	Y	Y	Y	Y	Y	Y	Y	Y	Y	Y
Mohammad	2020	Y	Y	Y	Y	Y	Y	Y	Y	Y	Y
Lucas	2021	Y	Y	Y	Y	Y	Y	Y	Y	Y	N
Yang	2020	Y	Y	Y	Y	N	Y	Y	Y	N	N
Tallegas	2019	N	N	Y	Y	N	N	N	Y	N	N
Nicolle	2019	N	N	Y	N	N	Y	Y	Y	N	N
Lam	2019	Y	Y	N	Y	Y	Y	Y	Y	N	N
Kerr	2013	Y	Y	Y	Y	Y	Y	Y	Y	N	N
Kanamori	2015	Y	Y	Y	Y	N	Y	Y	Y	N	Y
Internal Data	-	Y	Y	Y	Y	N	Y	Y	Y	Y	Y

**Table 2:** Patient Characteristics. A detailed breakdown of overall patient characteristics derived from raw patient data collected from included studies. Most patients fell into the 41–60 and 61–80-year-old age groups. Patients with metastatic disease (245, 34.22%) and locally recurrent disease (98, 35.51%) were well represented. In terms of final follow up status, patients were categorized as no evidence of disease (NED) (447, 60.41%), alive with disease (AWD) (47, 6.35%), dead of disease (DOD) (193, 26.08%), and dead of other cause/unknown (DO) (53, 7.16%).

	N	%
**Total**	1152	100.00%
**Age Group**		
<40	144	23.38%
41 to 60	232	37.66%
61 to 80	218	35.39%
>80	22	3.57%
**Sex**		
Male	395	56.83%
Female	300	43.17%
**Metastatic Disease**		
Y	245	34.22%
N	471	65.78%
**Local Recurrence**		
Y	98	35.51%
N	178	64.49%
**Final Disease Status**		
NED	447	60.41%
DOD	193	26.08%
AWD	47	6.35%
DO	53	7.16%

**Table 3:** Tumor Characteristics. A detailed breakdown of overall tumor characteristics derived from raw data collected from included studies. Grade 1 (300, 26.7%), grade 2 (503, 44.8%), grade 3 (101, 9.0%), and dedifferentiated tumors (220, 19.6%) were well represented in the set. The dataset was roughly balanced between IDH wild-type (WT) (545, 47.19%) and IDH mutant (610, 52.81%) tumors. Appendicular tumors (556, 54.09%) were slightly more common than axial tumors (472, 45.91%). The femur was the most common tumor site (254, 24.71%), and just over half of tumors were less than 10 cm in diameter (431, 58.9%).

	N	%
**Tumor Grade**		
I	300	26.69%
II	503	44.75%
III	101	8.99%
Dedifferentiated	220	19.57%
**IDH Status**		
Mutant	610	52.81%
WT	545	47.19%
**Axial vs. Appendicular**		
Appendicular	556	54.09%
Axial	472	45.91%
**Tumor Site**		
Chest Wall	74	7.20%
Clavicle	3	0.29%
Facial Bones	50	4.86%
Femur	254	24.71%
Fibula	19	1.85%
Foot/Ankle	25	2.43%
Hand/Lower Arm	45	4.38%
Humerus	121	11.77%
Pelvis	171	16.63%
Sacrum	10	0.97%
Scapula	30	2.92%
Shoulder	4	0.39%
Skull Bones	75	7.30%
Spine	32	3.11%
Tibia	55	5.35%
Tracheolaryngeal	60	5.84%
**Tumor Size (cm)**		
<5	171	23.39%
≥5 & <10	260	35.57%
≥10 & <15	168	22.98%
≥15	132	18.06%

**Table 4:** Sample Characteristics IDH Wild Type (WT) vs. Any IDH Mutation. A description of the sample based on IDH WT status and the presence of any IDH mutation (* indicates *p* < 0.05). Patients with IDH mutant tumors were more likely to be in the 61-–80-year-old age range (39.53% vs. 31.43%, *p* = 0.0354) and less likely to fall in the less-than-40 age range (17.94% vs. 28.57%, *p* = 0.0018) than patients with WT tumors. IDH mutant tumors were more likely to display dedifferentiation than WT tumors (26.42% vs. 11.66%, *p* < 0.0001). Patients with IDH mutant tumors were more likely to have died of disease at final follow up compared to their WT counterparts (28.72% vs. 21.09%, *p* = 0.0246). IDH mutant tumors were less likely to be associated with local recurrences than WT tumors (28.97% vs. 42.75%, *p* = 0.0169), and IDH mutant tumors were more likely to be found at appendicular sites than WT tumors (68.29% vs. 37.42%, *p* = 0.0312).

	IDH Mutant (n)	IDH Mutant (%)	WT (n)	WT (%)	*p* Value
**Whole Sample**	609	100.00%	543	100.00%	-
**Age Group**					
<40	54	17.94%	90	28.57%	0.0018 *
41 to 60	113	37.54%	119	37.78%	0.9517
61 to 80	119	39.53%	99	31.43%	0.0354 *
>80	15	4.98%	7	2.22%	0.8172
**Sex**					
Male	210	58.66%	182	54.49%	0.2689
Female	148	41.34%	152	45.51%	0.2689
**Tumor Grade**					
I	125	20.90%	175	33.46%	<0.0001 *
II	252	42.14%	249	47.61%	0.0661
III	63	10.54%	38	7.27%	0.0565
Dedifferentiated	158	26.42%	61	11.66%	<0.0001 *
**Final Disease Status**					
NED	277	57.23%	170	66.41%	0.0152 *
DOD	139	28.72%	54	21.09%	0.0246 *
AWD	28	5.79%	19	7.42%	0.3852
DO	40	8.26%	13	5.08%	0.1098
**Metastatic Disease**					
Y	159	35.73%	84	31.23%	0.2184
N	286	64.27%	185	68.77%	0.2184
**Local Recurrence**					
Y	42	28.97%	56	42.75%	0.0169 *
N	103	71.03%	75	57.25%	0.0169 *
**Axial vs. Appendicular**					
Appendicular	379	68.29%	177	37.42%	0.0312 *
Axial	176	31.71%	296	62.58%	0.0312 *

**Table 5:** Sample characteristics IDH 1 vs. IDH 2 mutations. A breakdown of the IDH mutant sample between IDH 1 and IDH 2 mutant tumors (* indicates *p* < 0.05). These data were not available in all studies, which limited our ability to include this variable in subsequent analyses. Patients with lower grade tumors (grade 1 and grade 2 chondrosarcoma) were more likely to have IDH 1 mutant tumors (23.97% vs. 11.38% in grade 1 (*p* = 0.0024), 44.06% vs. 32.53% in grade 2 (*p* = 0.021), respectively), while patients with dedifferentiated tumors were more likely to have IDH 2 mutant tumors (22.89% vs. 43.09%, *p* < 0.0001). Patients with appendicular disease were more likely to have IDH 2 mutant tumors (62.79% vs. 89.52%, *p* < 0.0001) and patients with axial disease were more likely to have IDH 1 mutant tumors (37.21% vs. 10.48%, *p* < 0.0001).

	IDH1 Mutant (n)	IDH1 Mutant (%)	IDH2 Mutant (n)	IDH2 Mutant (%)	*p* Value
**Whole Sample**	472	100.00%	125	100.00%	-
**Age Group**					
<40	46	19.91%	8	13.79%	0.285
41–60	87	37.66%	19	32.76%	0.4884
61–80	88	38.10%	26	44.83%	0.3483
>80	10	4.33%	5	8.62%	0.1877
**Sex**					
Male	161	58.33%	41	58.57%	0.9712
Female	115	41.67%	29	41.43%	0.9712
**Tumor Grade**					
I	111	23.97%	14	11.38%	0.0024 *
II	204	44.06%	40	32.52%	0.021 *
III	42	9.07%	16	13.01%	0.1937
Dedifferentiated	106	22.89%	53	43.09%	<0.0001 *
**Final Disease Status**					
NED	223	59.63%	48	48.98%	0.0578
DOD	109	29.14%	30	30.61%	0.7766
AWD	18	4.81%	4	4.08%	0.7599
DO	24	6.42%	16	16.33%	0.0017 *
**Metastatic Disease**					
Y	124	35.13%	35	38.04%	0.6032
N	229	64.87%	57	61.96%	0.6032
**Local Recurrence**					
Y	35	28.23%	5	26.32%	0.8629
N	89	71.77%	14	73.68%	0.8629
**Axial vs. Appendicular**					
Appendicular	275	62.79%	94	89.52%	<0.0001 *
Axial	163	37.21%	11	10.48%	<0.0001 *

**Table 6:** Multivariate Cox Proportional Hazards Model Results—Five-year OS vs. IDH Status and Dedifferentiated vs. Conventional Chondrosarcoma. The results of multivariate CPH models relating to five-year OS (* indicates *p* < 0.05). Patient sex was not a significant predictor of survival and is omitted from the results presented here. IDH mutation status correlated with OS in the full cohort (HR: 1.6, *p* = 0.03), however, this was driven by the dedifferentiated cohort. When analyzed separately, there was no correlation of IDH mutation status in the conventional chondrosarcoma group, but rather only in the dedifferentiated tumors, which was statistically significant (HR: 1.99, *p* = 0.02). Patient age (HR: 1.04, *p* < 0.005) and tumor grade (HR: 2.72, *p* < 0.005) were the only significant predictors of survival in patients with conventional chondrosarcoma.

**Total Sample**			
**Feature**	**HR**	**95% CI**	***p* Value**
Age	1.02	(1.01, 1.03)	0.01 *
Tumor Grade	3.23	(2.55, 4.09)	*p* < 0.005 *
Any IDH Mutation	1.65	(1.04, 2.61)	0.03 *
**Conventional Tumors**			
**Feature**	**HR**	**95% CI**	***p* Value**
Age	1.04	(1.01, 1.06)	*p* < 0.005 *
Tumor Grade	2.72	(1.46, 5.07)	*p* < 0.005 *
Any IDH Mutation	0.93	(0.41, 2.07)	0.85
**Dedifferentiated Tumors**			
**Feature**	**HR**	**95% CI**	***p* Value**
Age	1.01	(0.99, 1.03)	0.22
Any IDH Mutation	1.99	(1.10, 3.60)	0.02 *

**Table 7:** Multivariate Cox Proportional Hazards Model Results—OS vs. IDH Status and Dedifferentiated vs. Conventional Chondrosarcoma—High-grade vs. Low-grade Predictor. The results of multivariate CPH models relating to patient age, patient sex, tumor grade (high vs. low), and IDH status with five-year OS (* indicates *p* < 0.05). Again, patient sex was not a significant predictor of survival in this analysis and is omitted from the results presented here. Interestingly, the effect of IDH mutation in this analysis became even less apparent in the overall cohort and remained non-contributory in the conventional cohort. The presence of IDH mutation was only a significant predictor of OS in the dedifferentiated chondrosarcoma population (HR: 1.99, *p* = 0.02). As one might imagine, grading became a much more powerful predictor of survival in conventional chondrosarcoma when high-grade vs. low-grade designation was used (HR: 11.2, *p* = 0.02).

**Total Sample**			
**Feature**	**HR**	**95% CI**	***p* Value**
Age	1.03	(1.02, 1.04)	*p* < 0.005 *
High Grade	25.71	(3.56, 185.63)	*p* < 0.005 *
Any IDH Mutation	1.53	(0.98, 2.41)	0.06
**Conventional Tumors**			
**Feature**	**HR**	**95% CI**	***p* value**
Age	1.04	(1.01, 1.06)	*p* < 0.005 *
High Grade	11.2	(1.50, 83.52)	0.02 *
Any IDH Mutation	1.23	(0.58, 2.61)	0.6
**Dedifferentiated Tumors**			
**Feature**	**HR**	**95% CI**	***p* value**
Age	1.01	(0.99, 1.03)	0.22
Any IDH Mutation	1.99	(1.10, 3.60)	0.02 *

## Data Availability

Data are available upon request.
